# Dynamic Oil-in-Water Concentration Acquisition on a Pilot-Scaled Offshore Water-Oil Separation Facility

**DOI:** 10.3390/s17010124

**Published:** 2017-01-10

**Authors:** Petar Durdevic, Chitra S. Raju, Mads V. Bram, Dennis S. Hansen, Zhenyu Yang

**Affiliations:** 1 Department of Energy Technology, Aalborg University, Esbjerg 6700, Denmark; mvb@et.aau.dk (M.V.B.); dsh@et.aau.dk (D.S.H.); yang@et.aau.dk (Z.Y.); 2 Independent Consultant, Esbjerg 6700, Denmark; chitrasraju@gmail.com

**Keywords:** oil in water, oil and gas, offshore, dynamic, on-line monitoring, process control

## Abstract

This article is a feasibility study on using fluorescence-based oil-in-water (OiW) monitors for on-line dynamic efficiency measurement of a deoiling hydrocyclone. Dynamic measurements are crucial in the design and validation of dynamic models of the hydrocyclones, and to our knowledge, no dynamic OiW analysis of hydrocyclones has been carried out. Previous studies have extensively studied the steady state efficiency perspective of hydrocyclones, and have related them to different key parameters, such as the pressure drop ratio (PDR), inlet flow rate, and the flow-spilt. Through our study, we were able to measure the dynamics of the hydrocyclone’s efficiency (ϵ) response to step changes in the inlet flow rate with high accuracy. This is a breakthrough in the modelling, control, and monitoring of hydrocyclones.

## 1. Introduction

In the offshore Oil and Gas industry, instrumentation is kept at a minimum due to several factors, such as reliability, costs of installation, and difficulty of maintenance. Offshore installations are vastly complex and are tightly packed with equipment which require consistent feedback to ensure a satisfying performance. Due to the high costs and safety considerations, the industry avoids installing equipment and updating current control paradigms without concrete evidence that the new equipment can and will perform better and more reliably than the currently operating equipment and methods. Our work focuses on the deoiling hydrocyclone operation, where water and small concentrations of oil are separated, usually below 1% oil-in-water (OiW) concentrations [1]. The hydrocyclone separates oil from water by an enhanced gravity method, where the two phases are injected into a cylindrical chamber which—due to properties inherent in its design—induces the mixture to be spun into a vortex. This motion forces the oil droplets towards the centre of the cylindrical chamber due to the centripetal force, and forces the water towards the cylinder’s wall. There is a narrow exit on one side of the cylindrical chamber called the overflow through which the separated oil exits if the separation has been successful; i.e., if the forces were sufficient to force the oil droplets towards the centre. The funnel shape of the cylindrical chamber pushes the water close to the wall, and the water exits through an opening at the end of the funnel called the underflow. The funnel additionally acts to create a back pressure which ensures that some of the liquid—preferably only oil—is pushed through the overflow [2]. The obvious method for controlling such a system would be to measure and use the hydrocyclone’s efficiency (*ϵ*); see Equation (1) [1].

(1) $$\epsilon =1-\frac{C_{u}}{C_{i}}$$

where $C_{i}$ and $C_{u}$ is the OiW concentration in the inlet and underflow of the hydrocyclone, respectively. Yet the current control strategy for hydrocyclones does not consist of direct efficiency measurement, but instead is based on an indirect method, where a pressure drop ratio (PDR) measurement is used for control—refer to Equation (2) [3].

(2) $$PDR=\frac{p_{i}-p_{o}}{p_{i}-p_{u}}$$

where $p_{i}$, $p_{u}$ and $p_{o}$ are the pressures in the inlet, underflow and overflow respectively. The PDR control paradigm operates on the empirical evidence that the PDR is almost linearly proportional to the flow split ratio ($F_{s}$), refer to Equation (3) [4].

(3) $$F_{s}=\frac{F_{o}}{F_{i}}$$

where $F_{o}$ is the overflow flow rate and $F_{i}$ is the inlet flow rate. The flow split has further been empirically linked to the hydrocyclone’s efficiency [4]. The problem with using the PDR for *ϵ* control of the hydrocyclone is that these empirical relations are all made from a steady state perspective and for specific operating conditions. The reason for the common use of PDR is the superior reliability and precision of pressure transmitters compared to most existing technologies.

As far as we know, direct *ϵ* measurements have not been used as a feedback parameter for efficiency control of hydrocyclones on the North Sea Oil and Gas platforms. This is because a feedback control strategy based directly on the *ϵ* requires a reliable *ϵ* measurement with a sufficient sampling rate, but so far no such feedback transmitters are installed on current North Sea installations. Current installations rely on an offline OSPAR reference method ISO9377-2 [5] to measure the OiW concentration based on which the *ϵ* is calculated. The OSPAR reference method ISO9377-2 requires at least two samples per day to comply with government regulations, and to our knowledge, the samples are taken around three times per day. This low sampling rate has disadvantages, as it could be unrepresentative of the changes that occur through the day. This is not useful as a dynamic controller’s feedback parameter, as frequent and reliable samples and measurements are required for dynamic analysis of *ϵ*. The sampled measurements by the OSPAR reference method could, however, be used as key tuning parameters in a scheduled control paradigm, but this aspect is not considered in this work. Thus, in order to use *ϵ* as a feedback parameter, it is necessary to find a method of measuring the OiW concentration quickly and reliably.

The aim of this work was to investigate the possibility of using fluorescence-based technology to measure OiW concentrations online under dynamic conditions, such that these measurements could further be used to determine the *ϵ* of a pilot-scaled offshore deoiling facility. Our earlier work investigated the same OiW monitor—Turner Design TD-4100XDC (TD-4100), a commercially available OiW monitor manufactured by Turner Designs—for its offline steady state and dynamic response [6].

This work indicated that the TD-4100 performed well regarding steady state measurement. Thus, to investigate the performance of the TD-4100 under online dynamic conditions, the TD-4100 was installed on a pilot-scaled plant equipped with two full-sized offshore hydrocyclone liners. One drawback of using optical monitors is the possibility of fouling of the view-cell, which could lead to a drift in the measurement. This aspect has not been addressed in our current work, but in the case of real time applications, this could probably be avoided by using the non-contact falling stream flow cell found in TD-4100XD, where the media is not in contact with the view-cell.

The custom-built pilot-scaled plant (located at Aalborg University, Esbjerg campus, Esbjerg, Denmark) gives the flexibility to emulate realistic offshore scenarios, where parameters such as the volumetric inlet flow rate $F_{i}$ and the PDR can dynamically be varied in order to affect the *ϵ*. In addition, by using industrial liners provided by our partners (refer to Table 1), we were able to emulate real scenarios that occur on offshore oil and gas platforms. We measured the *ϵ* by measuring the concentration of oil in the inlet and in the underflow, and then calculated *ϵ* based on the previously introduced Equation (1). To validate the flow rate measurement, the pressure measurement was included and compared to the flow rate measurement.

Meldrum [3] initiated the investigation of hydrocyclone liners in their early stages of development; the work was done on a full-scale installation (the Murchison field), and involved the analysis of the correlation between $F_{i}$ and *ϵ*, $F_{s}$ and *ϵ*, and $F_{s}$ and PDR. The performance evaluation was done solely from the steady state perspective, and the results only proved the potential of the use of hydrocyclones in offshore installations. Similar findings were made in [1,4], where different hydrocyclone types were tested for their steady state performance. The study in [7] involved steady state analyses of a hydrocyclone liner, where investigations of the PDR, flow split, and *ϵ* were analysed on an in-house hydrocyclone set-up. The work done in [7] is an extension of [1,3,4], but it did not achieve high enough flow rates to achieve sufficient separation. In addition, no control-oriented dynamic models have been developed linking the PDR and *ϵ*, which could help in the development of model-based control techniques of the hydrocyclone’s efficiency. Although the hydrocyclone’s *ϵ* has been extensively researched (as mentioned earlier), it has been done only from the steady state perspective. The reason being that on-line, dynamic measurement of OiW is not straight-forward, due to several factors which are mentioned in the following paragraph.

First, the oil concentrations in the hydrocyclone are often varying, from as low as a few parts per million (PPM) to around 1000 PPM [8]. As the equipment’s PPM measurement is only approximately linear, it is calibrated to a certain operating range to assure a nearly linear response [6], and thus large variations in the concentration can result in measurement uncertainties. Second, the OiW equipment can rarely manage the high flows of the hydrocyclone installations, so they are installed instead on side streams with lower flow rates. This poses several difficulties, such as time delay due to the length of the connecting pipelines and the statistical possibility of misrepresentation of the actual flow as only a fraction of the flow enters the view cells for sampling [6]. Third, the inhomogeneous composition of crude oil poses many calibration issues, as one type of oil may require a different calibration curve than a slightly different type of oil [9]. Lastly, even though the OiW equipment has been studied for a long period of time—with the fluorescence-based instruments being the most widespread technique [9]—their use has been limited to monitoring. To our knowledge, they have not been tested as feedback transmitters under dynamic conditions, and verification of their precision still requires further evaluation—especially regarding their ability to measure dynamic changes.

The main achievement of this work was a successful measurement of the system efficiency *ϵ* using the two fluorescence OiW monitors (TD-4100), where dynamic changes in *ϵ* could be measured when the system was subjected to a changing $F_{i}$.

The article is structured as follows: Section 2 introduces materials, methods, and the experiment design, Section 3 explains the system’s operating conditions and the results, Section 4 discusses the results, and Section 5 concludes the article.

## 2. Materials and Methods

The pilot plant used for the experiments consists of a reservoir tank which has an attached pipeline and riser. The pipeline—which stretches 25 m in length horizontally—ends with a pipeline riser which raises the liquid 6 m up and onto a platform. The platform consists of a three-phase gravity separator and a deoiling hydrocyclone separator. The uniqueness of our pilot plant set-up is its versatility, where each of the mentioned subsystems can be decoupled and can operate individually. The system considered in this work only consists of the hydrocyclone and the reservoir tank. This is to isolate the hydrocyclone, as we wish to investigate the dynamic efficiency of this unit exclusively and to reduce the effects of the inherent dynamics of the other equipment that are not utilised in the current study.

**Figure 1:**
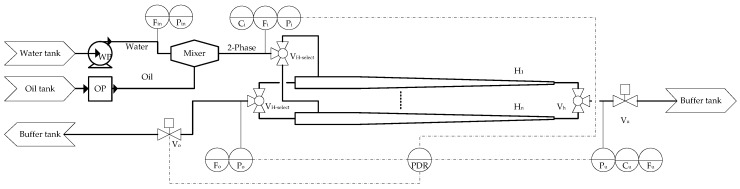
Sketched diagram of the plant, including the feeding system, the hydrocyclone array, and the transmitters used in the study. $C_{i}$, $C_{u}$: Turner Design TD-4100XDC (TD-4100) fluorescence monitors; $F_{in}$, $F_{i}$, $F_{u}$: electromagnetic flow transmitters; $F_{o}$: Coriolis flow transmitter; OP: oil pump; *P*: pressure transmitter; PDR: pressure drop ratio; *V*: valve; WP: water pump.

The pilot plant system that was used is illustrated in Figure 1, and the equipment involved is presented in Table 1.

### 2.1. Flow Transmitters

Electromagnetic flow transmitters (Rosemount 8732, Rosemount Inc., Shakopee, MN, USA and Bailey-Fischer-Porter 10DX4311C, ABB Ltd., Zurich, Switzerland) are used for flows with high water concentrations, as they are well suited for measuring flows of conductive material. The electromagnetic transmitters have the following tags: $F_{in}$, $F_{i}$, and $F_{u}$; these were placed at points where the oil concentration is less than 1%.

The measurement of multiphase flow (in this case, the two phases are oil and water) or non-conductive phase flow was done with Coriolis flow transmitters. A Coriolis flow transmitter (Micro-Motion Coriolis Elite CMFS010, Emmerson Micro Motion, Boulder, CO, USA) was placed at the hydrocyclone’s overflow, as this point has flows with a high oil concentration. The Coriolis flow transmitter has the tag $F_{o}$; refer to the diagram in Figure 1 and Table 1.

### 2.2. Pressure Transmitters

All the pressure transmitters used on the set-up were of the same type (Siemens Sitrans P200, Siemens, Munich, Germany), and use a piezo-resistive measuring cell with a ceramic diaphragm. This type of pressure transmitter has a high step response time of <5 ms. The pressure transmitters used have the following tags: $P_{in}$, $P_{i}$, $P_{u}$, $P_{o}$, and $P_{s}$; refer to the diagram in Figure 1 and Table 1. The PDR was calculated from the values collected from $P_{i}$, $P_{u}$, and $P_{o}$.

### 2.3. OiW Measurements

The OiW concentration was measured using the fluorescence monitors (Turner-Design TD-4100XDC, Turner-Design, San Jose, CA, USA), which detect the aromatics in the oil and through a calibration curve convert the relative fluorescence unit (RFU) to the related parts per million (PPM) value. The calibration procedure can be seen in our previous work; refer to article [6]. The equipment promises a refresh rate of 3 s and a detection range of 5 PPB–500 PPM, depending on the calibration [10]. Two TD-4100s were used, one at the hydrocyclone inlet, and one at the hydrocyclone outlet, with the tags $C_{i}$ and $C_{u}$.

### 2.4. Data Acquisition

The transmitters and actuators were connected to a series of I/O cards (NI PCI-6229, National Instruments, TX, USA) installed in a Simulink xPC Target real-time environment, linked through an Ethernet connection to a computer running Mathworks Simulink. The sampling frequency was kept constant for the entire set-up at 100 Hz, and all the data was stored on the computer. The system was oversampled to allow for high frequency transmitter extensions in the future. This set-up allows for versatile implementation of controller strategies directly in Simulink.

### 2.5. Materials

The oil and water mixture used for the tests was synthetic and was made of a mixture of tap water and mineral motor oil (ARDECA SAE30, NV Vroman, Vichte, Belgium). The mineral motor oil was chosen, as it is close to the viscosity of crude oil, it was the least purified oil available, and because the use of natural crude oil would pose a fire hazard. The water and oil mixture was kept at room temperature throughout the test, with an average temperature of 20.43 ${}^{\circ }$C. The tests were performed solely with the aforementioned liquids, as no additional chemicals were injected during the test, nor were any present in the buffer tanks.

### 2.6. Experiment Design

**Figure 2:**
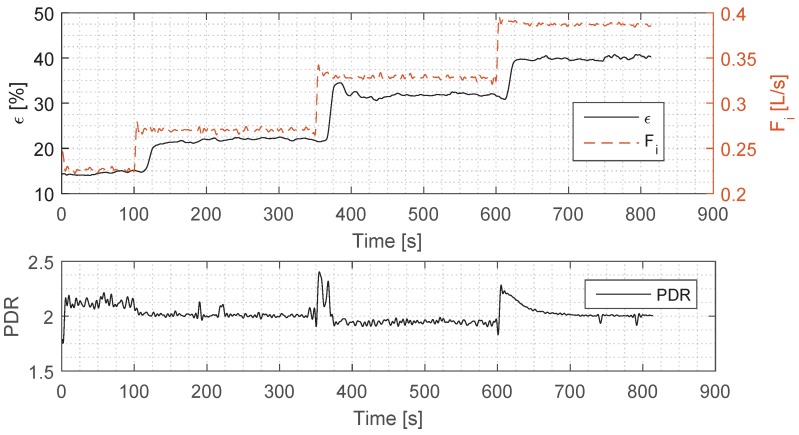
Experimental results: the (**top**) plot illustrates the calculated *ϵ* and $F_{i}$; The (**bottom**) plot illustrates the PDR.

The aim of the experiment was to investigate if the fluorescence-based equipment could track the dynamic changes in OiW concentrations at the hydrocyclone inlet and outlet. In order to achieve an observable response from the *ϵ* measurement, the system needed sufficient excitation. To achieve this, the inlet flow rate was stepped between four different values. To assure a consistent volumetric flow rate, the pump was controlled using a Proportional-Integral-Derivative (PID) flow controller with the $F_{in}$ flow measurement as the feedback parameter. The chosen $F_{i}$ step inputs were based on an empirical investigation of the influence of the flow rates on the *ϵ*. The oil was injected into the mixer where the shear forces dispersed the oil into the water. The flow from the oil pump was controlled using the built-in flow controller. The PDR was kept stable using a PID controller to reduce its impact on the system performance; the resulting PDR that was measured is illustrated in the bottom plot of Figure 2 together with $F_{s}$. The valve used in the control of the PDR has an inbuilt PID controller which aims at achieving the desired valve opening position. The PID controller was tuned using a trial and error method until a satisfactory performance was achieved; i.e., the dynamics of the valve are faster than the other dynamics of the system, such as the pressures and the flows. Operating conditions for this experiment are shown in Table 2. The collected data was filtered using a low-pass filter with a 0.2 Hz cut frequency to reduce unwanted sensor and measurement noise.

## 3. Results

### 3.1. Operating Conditions

The comparison of $F_{i}$ and the system efficiency represented by *ϵ* is shown in the top plot of Figure 2. The requirement for good results is that $F_{i}$ has a step input amplitude which should produce a corresponding step deviation in *ϵ*, and as seen from the results, this has been achieved.

Secondly, in order to remove any steady state bias from $F_{i}$, it is crucial that $F_{i}$ closely tracks its reference. The $F_{i}$ has an average standard deviation from its mean of σ=0.0014 L/s, which is a small deviation considering that the mean of the three individual steps is 0.2704 L/s, 0.3289 L/s, and 0.3871 L/s, and $F_{i}$ is thus considered suited for the experiment. The PDR—plotted in the bottom plot of Figure 2—stays close to its reference of 2, with small deviations from steady state around the step inputs. During the second step, the PDR is offset with 0.05 from the PDR set-point, which is caused by the internal valve hysteresis. Based on previous experience, small deviations in the PDR do not have much significance on system *ϵ*, as long as the PDR is kept within a safe boundary; therefore the PDR that was used is considered well-suited for the experiments.

### 3.2. Results

The impact of each step input in $F_{i}$ on *ϵ* is presented in Table 3, which shows the time of each individual step, steady state mean amplitude, steady state standard deviation, percentile increase from previous step’s steady state mean to current steady state mean, percentile deviation of the two signals’ step increase, and time delay between the two signals’ response and rise-time, where the rise-time is the time from which the step goes from 10% to 90% of its steady state value [11]. Table 3 also includes the percentile deviation of the two signals’ rise-time and the percentile overshoot of the signal, which is measured for the largest value’s percentile deviation from the new steady state mean. *ϵ* reacts to every step input of $F_{i}$ with an approximate time delay of 10 s. The response of $F_{i}$ is consistent with a rise-time between 1.83 s and 1.96 s, and a steady state mean increase of roughly 20%. The response of *ϵ* to the $F_{i}$ step input is not as consistent, where a steady state amplitude response of *ϵ* decreases for each step: 25.76 %, 20.18 %, and 6.49 % for the first, second, and the third step respectively.

**Figure 3:**
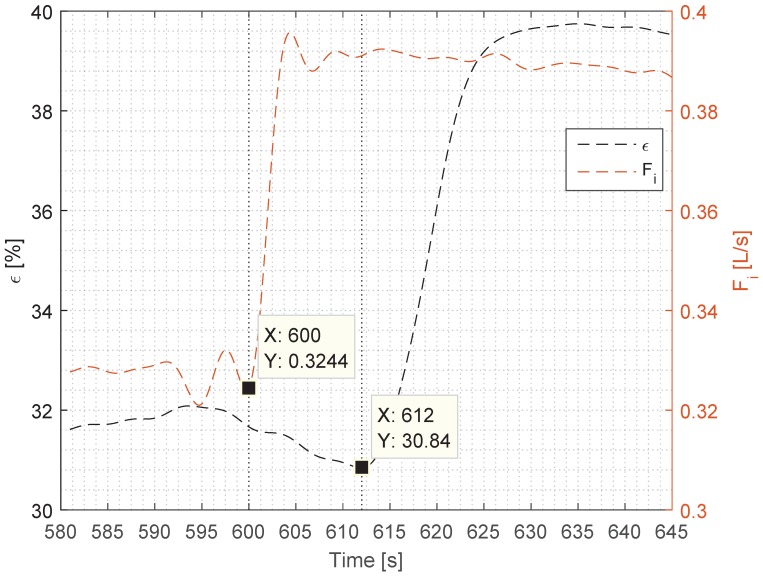
Zoomed view of the third step from Figure 2, illustrating the dynamic behaviour of *ϵ* and $F_{i}$. The dotted lines indicate the time of step input of $F_{i}$ and the approximate step response of *ϵ*.

To analyse the dynamics of the two signals in further detail, the signals from the third step are enlarged and shown in Figure 3. From this plot, the delay in the rise time of *ϵ* is evident, and by analysing the rise-time, it was found to be 7.9 s for *ϵ* and 1.96 s for $F_{i}$, which makes $F_{i}$ four times faster. This is also observable in the plot, where the trend of *ϵ* is less steep. A rise-time offset was consistently measured in the other steps, where it was slightly shorter in the second step, as *ϵ* exhibited an overshoot of 2.8% when compared to virtually no overshoot in step one, and an insignificant overshoot in step three. The overshoot in *ϵ* is consistent with an overshoot in $F_{i}$, which at this point is 0.0134 L/s compared to the overshoot value of 0.089 L/s and 0.086 L/s for the first and the third steps, respectively, thus making the second step’s overshoot ≈65% larger than the other two steps’ overshoot. The overshoot also means that the settling time for *ϵ* changes slightly, reaching its steady state within ≈80 s, ≈110 s, and ≈20 s for the first, second, and the third steps, respectively. The rise-time regarding *ϵ* in step one is slow compared to step two and step three, where the rise-time of $F_{i}$ is 1125.68% faster.

**Figure 4:**
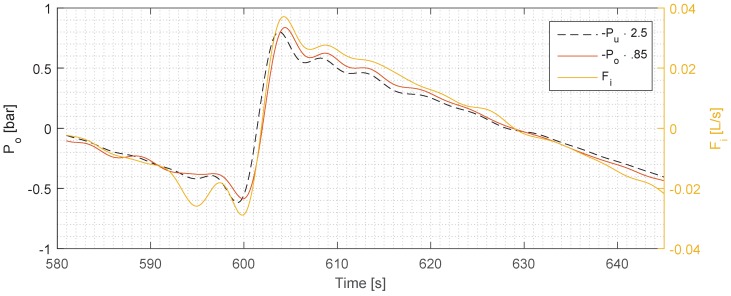
Comparison of $F_{i}$, $P_{u}$, and $P_{o}$.

**Figure 5:**
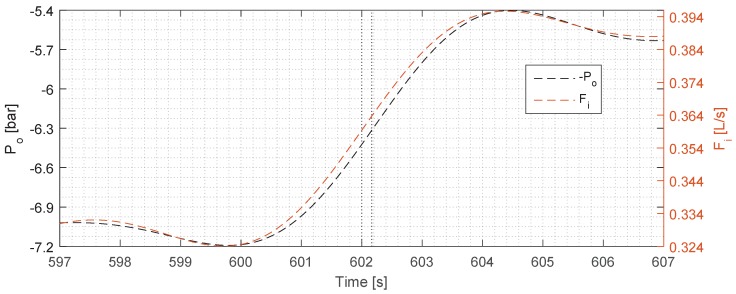
Comparison of $F_{i}$ and $P_{o}$; the dotted lines illustrate the delay between the two measurements, measured to be 0.165 s.

To confirm the validity of the $F_{i}$, it is compared to the pressure measurement, and if the measurements have consistent dynamics and steady state behaviour, $F_{i}$ can be considered valid. The result is shown in Figure 4, where the two pressures ($P_{u}$ and $P_{o}$) are chosen. These two pressures are the ones that get affected most by a change in $F_{i}$, in comparison to Pi, due to the back pressure over the two valves $V_{u}$ and $V_{o}$, which are located directly downstream of $P_{u}$ and $P_{o}$. The values are normalized, and their gain is adjusted to fit them on top of each other for easier comparison of the time delays. The step delay regarding the $F_{i}$ and $P_{o}$ is less than 0.1 s, and if $F_{i}$ and $P_{o}$ are compared, the delay is ≈0.45 s with a close to identical rise-time for all the measurements. A comparison of the rise-time of $F_{i}$ and $P_{o}$ is shown in Figure 5.

## 4. Discussion

The goal of the study was to analyse the ability of the TD-4100 to measure the dynamic changes in the hydrocyclone’s *ϵ*, where the changes were created by incrementing the inlet flow rate $F_{i}$ several times. The increments had to be sufficient to cause *ϵ* to change significantly enough to be measured, and this was achieved as *ϵ* responds to every step input of $F_{i}$, as seen in the top plot of Figure 2. In addition, the $F_{i}$ measurement was required to remain at a consistent steady state value; although small variations in the steady state were observed, the average coefficient of variation was <1%, indicating a high signal-to-noise ratio (SNR). The fluctuations were caused by small variations in the control signal to the pump being discretised by the pump frequency converter, and the discretisation of the flow measurement used for the pump controller feedback signal. Thus, the $F_{i}$ could be used as an excitation signal (due to the high SNR), without considerable influence on the dynamics of *ϵ*.

The $F_{i}$ step input was observed to have a direct impact on the *ϵ*, with a consistent delay of ≈10 s at each step. There are several factors which could contribute to this phenomenon; the first being the positioning of the TD-4100. The TD-4100 was connected on a side stream to the inlet and to the underflow of the hydrocyclone with pressure hoses, each of which were 3 m long and of a smaller diameter than the inlet and the underflow. The length of the hoses and the reduction in diameter (which introduces a pressure drop) affects the flow of the liquid, thus affecting the time it takes for the fluid to reach the equipment from the main line. Shorter hose connections to the equipment could reduce the delay of liquid entering the test chamber. The second reason is the response time of the TD-4100, which is estimated to be 3 s by the manufacturer [10]. It is expected that the delay is solely caused by the two mentioned reasons, but further research will investigate what causes this consistent delay.

The rise-time delay (as seen in Figure 3) has a more complicated explanation than the time delay, and we assume that it was caused by the dynamics of the separation, and our future work will aim to uncover the exact cause for it. Judging from the top plot in Figure 2, the rise-time—and thus the tangential of the *ϵ*—is similar for all three steps, which means that the TD-4100 is measuring consistently each time. We can observe from the overshoot in the second step—which occurs both in $F_{i}$ and *ϵ*—that the TD-4100 is able to track the dynamic behaviour of *ϵ*. As in the first and the second step where $F_{i}$ has considerably less overshoot, *ϵ* follows suit with no overshoot. Due to the complexity of the separation dynamics inside the hydrocyclone, it is hard to predict the exact behaviour of *ϵ*, and thus predict the outcome. However, our measurements do uncover a consistent relationship between $F_{i}$ and *ϵ*, which follows the theory of droplet separation formulated by Stokes’ law [12]. To validate our $F_{i}$ measurement, we have used the pressure measurements as a comparison, and the two measurements agree well with each other, which increases the validity of the flow measurements.

## 5. Conclusions

Our conclusion is thus that the fluorescence-based measurement monitor (the TD-4100) can successfully measure dynamic response of the hydrocyclone’s *ϵ*. In addition, the steady state and dynamic measurements of *ϵ* were consistent, and responded to the flow input in accordance to established laws of physics. The time delay and slower rise-time phenomena in the *ϵ* measurement—although a common effect in such systems—requires further investigation.

We propose that additional instruments be placed in series with the current instruments to enable further validation of the TD-4100. In addition, a reliability study of the fluorescence monitors should be done to evaluate their performance in a long term perspective. Finally, a different type of equipment should be introduced, preferably based on a different sensing paradigm, as an additional validation of the OiW measurement by the TD-4100.

## Figures and Tables

**Table 1:** Description of equipment used for the hydrocyclone set-up.

Name	Type	Description	Range/Size
WP	Grundfos CRNE 3	Centrifugal water feed pump	1 L/s at 162.7 m, max 25 bar
OP	Grundfos DDA	Mechanically actuated diaphragm oil feed pump	$(1.94\times 10^{-7}$ −0.0022) g/s, max 16 bar
H${}_{n}$	Vortoil liner	Up to two industrial hydrocyclone liners	1.4”
$P_{in,i,u,o,s}$	Siemens Sitrans P200	Piezo-resistive pressure measuring cell	(0–16) bar
$F_{in}$	Rosemount 8732	Electromagnetic flow transmitter	DN50 (0–25.966) L/s @ 12 m/s
$F_{i,u}$	Bailey-Fischer-Porter 10DX4311C	Electromagnetic flow transmitter	DN15 (0–1.64034) L/s
$F_{o}$	Micro-Motion Coriolis Elite (CMFS010)	Coriolis flow transmitter	DN10 $(1.389\times 10^{-5}$ −0.0033) L/s
Mixer	In-house-designed	Venturi based mixer	DN50
$C_{i,u}$	Turner-Design TD-4100XDC	Fluorescence measurement OiW monitor	(5 PPB–500 PPM)
$V_{u,o}$	Bürkert 2301 + 8696	Globe valve	$V_{o}=3$ mm $V_{u}=15$ mm

**Table 2:** Experimental operating conditions.

Parameters	Set-Points	Units
PDR	≈2	$\frac{\Delta p_{u}}{\Delta p_{o}}$
$F_{in}$	0.22−0.27−0.33−0.39	[L/s]
$P_{in}$	≈9.5	[bar]
$C_{i}$	≈400	[PPM]

**Table 3:** Step response and steady state analysis of *ϵ* and $F_{i}$; for more details, refer to Section 3.2.

Signal-Name	Step-Time	Steady State Mean (Steady State Standard Deviation)	Increase from Previous Mean	Deviation of $F_{i}$ from *ϵ* Mean	Step Delay	Rise-Time	Δ Rise-Time	Overshoot
**Initialisation**								
$F_{i}$	-	0.2264 L/s (0.0014) L/s	-	-	-	-	-	-
*ϵ*	-	0.1444 PPM (0.0033) PPM	-	-	-	-	-	-
**Step 1**								
$F_{i}$	100 s	0.2704 L/s (0.0029) L/s	119.43%	25.76%	-	1.83 s	1125.68%	3.2943%
*ϵ*	≈110 s	0.2169 PPM (0.0020) PPM	150.2%	-	≈10 s	20.6 s	-	0%
**Step 2**								
$F_{i}$	350 s	0.3289 L/s (0.0013) L/s	121.67%	20.18%	-	1.87 s	263.1%	4.0729%
*ϵ*	≈360 s	0.3171 PPM (0.0023) PPM	146.22%	-	≈10 s	4.92 s	-	8.8345%
**Step 3**								
$F_{i}$	600 s	0.3871 L/s (0.001) L/s	117.68%	6.4931%	-	41.96 s	403.06%	2.2187%
*ϵ*	≈612 s	0.3974 PPM (0.0037) PPM	125.32 %	-	≈12 s	7.9 s	-	0%

## Abbreviations

The following abbreviations are used in this manuscript:

PDR	Pressure Drop Ratio
OiW	Oil in Water
PPM	Parts Per Million
RFU	Relative Fluorescence Unit
SNR	Signal to noise ratio
PID	Proportional–Integral–Derivative Controller
